# 

**Published:** 1894-08

**Authors:** 


					﻿Vol XIV.
AUGUST, 1894.
NO. 8.
THE
[Iomœopathic physician,
A Monthly Journal of Homoeopathic Materia Medica
and Clinical Medicine.
“ He is a freeman whom truth makes frpcan.fi all are s	de'*
“ Seek the Truth : come uhencj{^\^y, cost w	u-ai."
ME 4. L).
PHILADELPHIA :
TTo.1231 LOCUST STREET.
London:
ALFRED HEATH A CO., 8olk Agents rob Great Britain,
114 Ebury Street, Eaton Square.
‘Yearly Subscription, $2.60, invariably in advance. Sinyle numbers^ 26 cte
Kntered at tbe Philadelphia PmvOAcc a* Seonod-Claaa Mai) Mauer.
				

